# Supplementary material for: Refining Stereotaxic Neurosurgery Techniques and Welfare Assessment for Long-Term Intracerebroventricular Device Implantation in Rodents
Source: Animals (Basel). 2023 Aug 14;13(16):2627. doi: 10.3390/ani13162627 (PMC10452028; doi:10.3390/ani13162627)
Supplement: Supplementary file 1 [file animals-13-02627-s001.zip › animals-2551067-supplementary.pdf]

Supplementary Material: Refining Stereotaxic Neurosurgery Techniques and Welfare Assessment for Long-term Intracerebroventricular Device Implantation in Rodents (Pérez-Martín, *et al.*, 2023)

This document includes the following Supplementary Material:

- Table S1: Overview of complications observed in the animals employed in our study.
- Figure S1: WT *versus* APP comparisons for body weight parameter at W-1, W3 and W8.
- Figure S2: WT *versus* APP comparisons for welfare assessment score parameter at W-1, W3 and W8.
- Figure S3: Detail of the percentage spent in open *versus* closed area in Elevated Zero Maze at W-1, W3 and W8.
- Supplementary Information S1: Step-by-step protocol for stereotaxic surgery procedure.
- Supplementary Information S2: Postoperative regimen of analgesic, anti-inflammatory and antibiotic treatment.
- Supplementary Information S3: Procedure and Postoperative monitoring form.

-Table S1-

Table S1. Overview of complications observed in the animals employed in our study.

Animal ID	Device	Genotype	Time Implanted	Complications	H.E.
169	None	WT	N/A	None	No
275	None	WT	N/A	None	No
287	None	WT	N/A	None	No
289	None	WT	N/A	None	No
335	None	WT	N/A	None	No
344	None	WT	N/A	None	No
487	None	WT	N/A	None	No
488	None	WT	N/A	None	No
492	None	WT	N/A	None	No
523	None	WT	N/A	None	No
160	None	APP	N/A	None	No
274	None	APP	N/A	None	No
276	None	APP	N/A	None	No
333	None	APP	N/A	None	No
348	None	APP	N/A	None	No
538	None	APP	N/A	None	No
539	None	APP	N/A	None	No
547	None	APP	N/A	None	No
548	None	APP	N/A	None	No
549	None	APP	N/A	None	No
59	Original	WT	48 hours	Hypolocomotion and lethargy, cannula detached	Yes (W0)
110	Original	WT	72 hours	Hypolocomotion and lethargy (W0)	Yes (W0)
17	Original	WT	3 weeks	Open wound and cannula detached (W3)	Yes (W3)
18	Original	WT	3 weeks	Loosen stitches and cannula detached (W3)	No
19	Original	WT	3 weeks	Loosen stitches (W3)	No
20	Original	WT	3 weeks	Open wound (W3)	Yes (W3)
38	Original	WT	3 weeks	Cannula detached (W3)	Yes (W3)
105	Original	WT	3 weeks	Open wound, reoperated (W1)	Yes (W3)
109	Original	WT	3 weeks	Open wound, reoperated (W2)	No
106	Original	APP	48 hours	Hypolocomotion and lethargy (W0)	Yes (W0)
36	Original	APP	1 week	Hypolocomotion and lethargy (W1)	Yes (W1)
117	Original	APP	2 weeks	Tremor and lethargy	Yes (W2)
34	Original	APP	3 weeks	None	No
35	Original	APP	3 weeks	None	No
39	Original	APP	3 weeks	None	No
107	Original	APP	3 weeks	None	No
108	Original	APP	3 weeks	None	No
116	Original	APP	3 weeks	Open wound, reoperated (W2)	No
122	Original	APP	3 weeks	Open wound, reoperated (W1 and W2)	Yes (W3)
331	Miniaturized	WT	8 weeks	Disconnection Tube-Device (W3)	No
349	Miniaturized	WT	8 weeks	Disconnection Tube-Cannula, reoperated (W5)	No
161	Miniaturized	WT	9 weeks	None	No
273	Miniaturized	APP	3 weeks	Cannula detached (W3)	Yes (W3)
277	Miniaturized	APP	8 weeks	None	No
285	Miniaturized	APP	8 weeks	None	No
286	Miniaturized	APP	8 weeks	None	No
334	Miniaturized	APP	8 weeks	Disconnection Tube-Device (W8)	No
336	Miniaturized	APP	8 weeks	None	No
345	Miniaturized	APP	8 weeks	Disconnected Tube-Device (W2)	No
346	Miniaturized	APP	8 weeks	Broken connecting tube (W8)	No
148	Miniaturized	APP	9 weeks	None	No
162	Miniaturized	APP	9 weeks	None	No

502	Alzet 1004	WT	4 weeks	None	No
503	Alzet 1004	WT	4 weeks	None	No
504	Alzet 1004	WT	4 weeks	None	No
505	Alzet 1004	WT	4 weeks	None	No
506	Alzet 1004	WT	4 weeks	None	No
516	Alzet 1004	WT	4 weeks	None	No
518	Alzet 1004	WT	4 weeks	None	No
522	Alzet 1004	WT	4 weeks	None	No
533	Alzet 1004	WT	4 weeks	None	No
536	Alzet 1004	WT	4 weeks	None	No
510	Alzet 1004	APP	1 week	Vestibulocochlear syndrome (W1)	Yes (W1)
489	Alzet 1004	APP	4 weeks	None	No
490	Alzet 1004	APP	4 weeks	None	No
491	Alzet 1004	APP	4 weeks	None	No
493	Alzet 1004	APP	4 weeks	None	No
511	Alzet 1004	APP	4 weeks	None	No
512	Alzet 1004	APP	4 weeks	None	No
532	Alzet 1004	APP	4 weeks	None	No
534	Alzet 1004	APP	4 weeks	None	No
535	Alzet 1004	APP	4 weeks	None	No

Legend: H.E., humane endpoint; N/A, not applicable; W, week; WT, wild type.

Note that most of the complications reported with the miniaturized device and Alzet pump are not related to the surgical procedure itself, but mainly to issues in the device components.

-Figure S1-

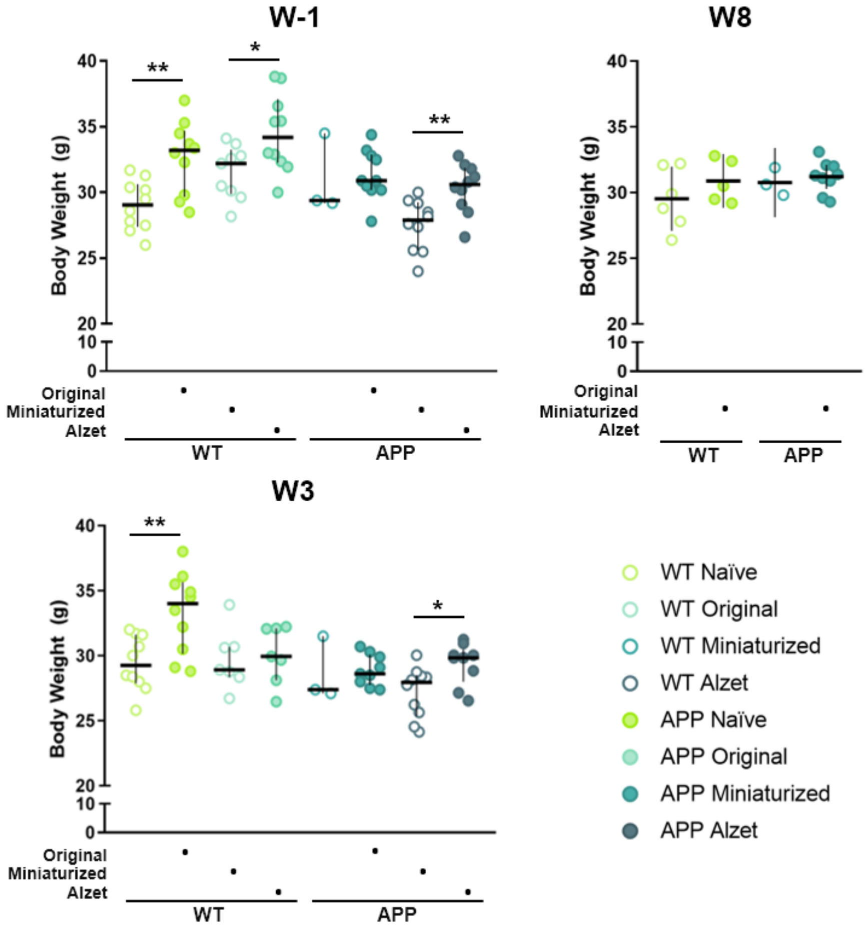

Figure S1. WT *vs.* APP comparisons for body weight parameter at W-1, W3 and W8. Specific sample sizes for each experimental group in Table 1. Mann-Whitney U test for WT *vs.* APP comparisons in all the experimental groups and timepoints analyzed. * $p < 0.05$; ** $p < 0.01$. W, week.

-Figure S2-

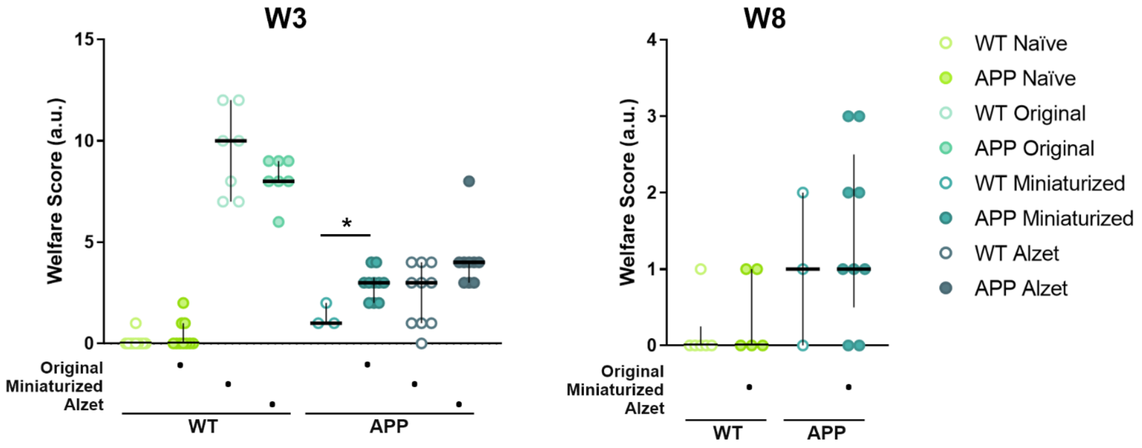

Figure S2. WT *vs.* APP comparisons for welfare assessment score parameter at W-1, W3 and W8. Specific sample sizes for each experimental group in Table 1. Mann-Whitney U test for WT vs. APP comparisons in all the experimental groups and timepoints analyzed. * $p < 0.05$. W, week.

-Figure S3-

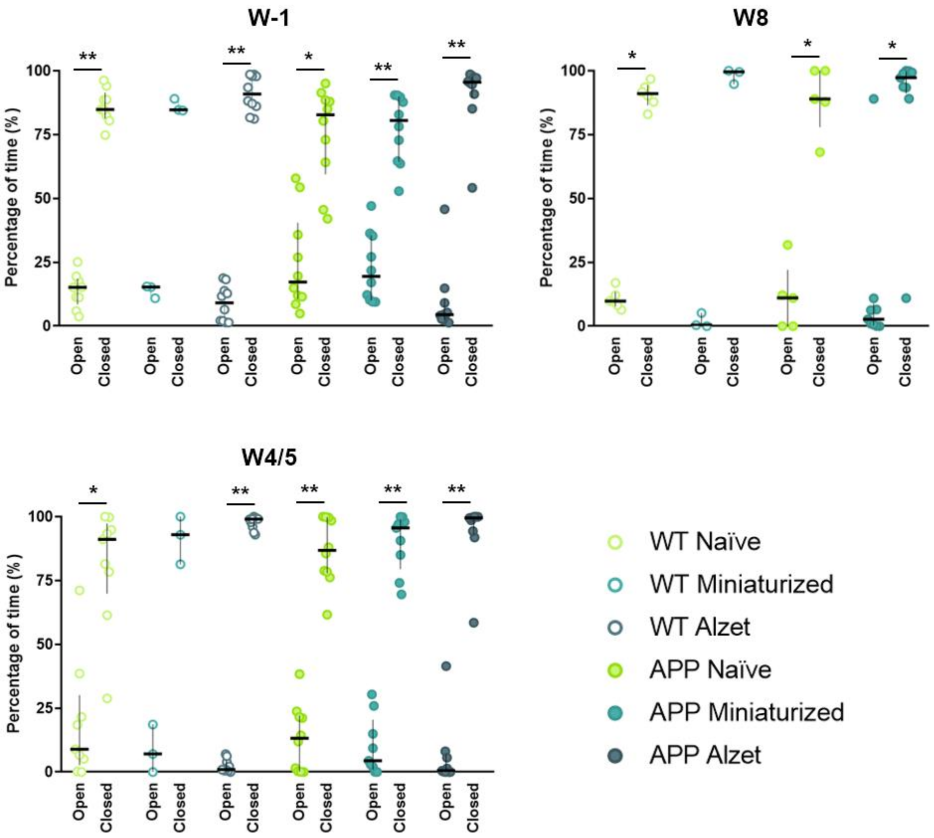

Figure S3. Detail of the percentage spent in open *vs*. closed area in Elevated Zero Maze at W-1, W3 and W8. Specific *n* sample sizes for each experimental group in Table 1. Wilcoxon test for all comparisons in all the experimental groups and timepoints analyzed. * $p < 0.05$, ** $p < 0.01$. W, week.

-Supplementary Information S1-

DEVICE IMPLANTATION SURGERY AND POSTOPERATIVE CARE PROTOCOL

I. MATERIAL AND EQUIPMENT REQUIRED

Equipment/Material/Product	Company	Reference
ALZET Brain Infusion Kit III	*ALZET*	0008851
Hamilton 805 RN Series 50 µl Syringe	*Hamilton Company*	7644-01
Digital Stereotaxic Apparatus for small rodents	*Harvard Apparatus*	HV751822
Probe holder, corner clamp	*Harvard Apparatus*	75-1870
Cannula holder 1	*ALZET*	0008860
Leica Stereomicroscope S9i	*Leica Microsystems*	10450816
LED Fiber Optic Dual Gooseneck Light Microscope Illuminator	*Microscope Central*	76312
Heating pad for rodents	-	-
Small recovery chamber or warming cabinet (ICU Vetario S50)	*Kanalvet*	MO1001235
UV Led Curing Bestled Lamp	*Bestdent*	LUX E
Aesculap Isis Rodent Shaver	*Aesculap Schermaschinen*	GT421
Cicastick Suturvet (Cyanoacrylate tissue adhesive)	*Chemical Iberica*	02960-H
Transbond XT 4 (UV Light Cure Orthodontic Adhesive)	*3M UNITEK*	712-035
Ethanol 70% (v/v)	-	-
Sterile Saline Solution 0.9% (w/v) NaCl	-	-
Povidone-iodine diluted in saline solution (1:1)	-	-
IsoFlo 100% (v/v) vapor inhalation liquid (Isoflurane)	*Zoetis Spain*	E3NTI100
Lubrithal Eye Gel	*Dechra*	
EMLA 25 mg/g + 25 mg/g Lidocaine and Prilocaine cream	*Aspen Pharma Trading*	679290.2
Fucidine 20 mg/g cream (Fusidic acid)	*Laboratorios Leo Pharma*	999794.6
Non-Absorbable Polypropylene Sutures 5-0	*Atramat*	P-PE1665
Sterile cotton swabs and paper wipes	-	-
Surgical instruments: scissors, tweezers, scraper, forceps, bone nipper, needles for craniotomy, needle holder…etc.		

II. PRE-SURGICAL PROCEDURE

Reservoir filling

Devices must be filled with the therapeutic agent/drug dissolved in the vehicle solution (artificial cerebrospinal fluid, aCSF) on the day of surgery, under sterile conditions.

The solution should be slowly introduced into the end of the reservoir with a 50 µl Hamilton Syringe taking care not to come out of the metal plate surrounding the reservoir. The volume of the reservoir is 50 µl while the total volume of the device is around 400 µl depending on the catheter length.

Note: It is important to confirm that the catheter is not blocked and that there are no bubbles inside the device. This can be done by adding vehicle solution until a drop appears at the end of the cannula.

Drug preparation

Analgesic, anti-inflammatory, and antibiotic drugs must be prepared freshly and under sterile conditions on the same day of the surgery to avoid drug degradation/contamination.

Note: After use on the day of surgery, drug dilutions last for 1 month if stored in a sterile vial at +4 °C.

Surgical bench set up and pre-surgical treatment

1. Thoroughly clean the working field area with 70% (v/v) ethanol and place the pre-sterilized surgical material and equipment.
2. Calibrate the stereotaxic frame as indicated by the supplier and turn out the heating pad.
3. 10 minutes before surgery, place the mice inside the warming cabinet (it helps to maintain vital signs during anesthesia and improves post-surgical recovery).
4. Weight and record the mouse body weight. Then, administer the pre-surgery treatment (Buprenorphine, enrofloxacin and saline solution).
5. Fill the anesthetic chamber with 4% (v/v) Isoflurane (IsoFlo) and 2.5% (v/v) O_2 and place the mouse inside until it is fully anesthetized (animal is not moving, its breathing rate decreases, and pedal withdrawal and tail pinch reflexes are absent).
6. Lightly shave the cephalic area where the surgery is to be performed.
7. Remove the white tip of the ALZET Cannula from the device and add an extra disc to achieve the desired depth (2.25 mm) when inserting the cannula into the brain. A special tissue glue (Cicastick Suturvet) can be used to fix the disc.

Important note: During the surgery, mouse breathing, and level of anesthesia must be checked constantly: if breathing rate increases, anesthesia may not be enough, increase isoflurane level; by contrast if breathing rate decreases isoflurane level should be decreased to avoid the animal die.

III. DEVICE IMPLANTATION SURGERY

8. Affix the mouse in the bite bar of the stereotaxic frame equipped with an inhalation mask for small rodents and decrease Isoflurane level to 2% (v/v).
9. Apply ophthalmic ointment (Lubrithal) with cotton tips to prevent eyes from drying out during the surgery (reapply if needed until the surgery is complete).
10. Apply local anesthetic cream in the external auditory canal before securing the head to the frame using the ear bars. To place ear-bars correctly find the zygomatic arch and insert the bars into the region just behind the arches, feeling the hardness of the skull. Bars should be fixed but not penetrating the ear canals (since the mouse can die). Check there is not movement of the mouse's head.
11. Clean surgery area with 70% (v/v) ethanol and Povidone-iodine (inside to outside circles).
12. Make an "U"-shaped incision on the back of the neck and pull the skin to the front to expose the skull and visualize the craniometric points *bregma* and *lambda*. During the surgery, apply sterile saline solution with a cotton swap on the skin to avoid drying out and/or necrosis.
13. Scrape the periosteum to the edges of the incision with a scrapper and dry the skull surface with a cotton stick moistened with 70% (v/v) ethanol.
14. Align the skull to standard stereotaxic coordinates:
    - Insert a fine-point needle (or epidural needle) in the probe holder attached to the stereotaxic manipulator arm.
    - Identify Bregma and Lambda on the skull and position the needle in bregma touching the skull. Set the coordinates to zero (Antero-posterior: AP or Y; medio-lateral: ML o X; dorso-ventral: DV or Z) in the digital display.
    - Move the needle far from skull in AP (y) to find lambda, and check Z-value. A difference less than 50 µm is allowed (0.05 in the digital display).
    - Move the height of the nose clamp if necessary.
    - Position the needle in AP: -2.0 and ML: ±2 to check if they have the same Z-value (or dorsoventral value). A difference less than 50 um is allowed (0.05 in the digital display)

    For bigger differences, reposition the mouse's head and recheck the coordinates again.
15. Set the specific coordinates where the cannula will be inserted in the left lateral ventricle (in this article: AP -1.26 mm, ML -0.7 mm) and slightly drill the skull without perforate. Clean the indentation area with saline and 70% (v/v) ethanol.
16. Take a pair of blunt scissors and slide under the skin from the neck incision backwards to make a pocket slightly larger than the size of the device.
17. Introduce the device so that the refilling window of the reservoir remains accessible, cut the catheter to the desired length, and reattach the cannula. Make sure that the cannula fits tightly to the catheter.
18. With the help of an insulin syringe or a small scalpel a craniotomy will be performed at the previously marked coordinates (refer to point 15). Take special care to avoid hemorrhages or damage to the brain surface. Clean the area with a cotton swap moistened in 70% (v/v) ethanol.
19. Attach the cannula holder (Alzet) in the stereotaxic manipulator arm and place the cannula inside the tip. Add Cicastick tissue glue with a small brush at the base of the cannula that will contact the skull surface and drive the cannula until the specific coordinates of the craniotomy with the manipulator arm.
20. Slowly deepen the cannula holder until the cannula base contacts the surface of the skull and wait two minutes for the glue to dry.

21. In the meantime, spread a thin layer of UV light-curing composite (Transbond XT 4) around the base of the cannula forming a ring and cure with UV light (Bestdent). It is recommended to wear UV protection glasses and to turn off the LED light lamp used for the surgery since the composite resin employed is photosensitive.
22. Carefully remove the cannula from the holder and cut off the upper tip with a bone nipper. In this step it is crucial to ensure that the cannula is well fixed to the skull, otherwise it will detach.
23. Suture the incision with 5-0 polypropylene thread to ensure proper healing. It is highly recommended to make a higher number of stitches in the maximum tension area near the catheter.
24. Apply povidone iodine and topical antibiotic cream (Fucidine) over the suture with a sterile cotton swap.
25. Carefully cut the tips of the nails both hind and forelimbs to prevent the animals from pulling them off in the weeks following the surgery.
26. Remove the animal from stereotaxic frame and inject SC meloxicam and saline solution.
27. Place each mouse individually in a clean cage with gel food on the floor and put it inside the recovery or ICU chamber (28-30 °C). Wait for the mouse to fully recover and wake up.

IV. POST-SURGICAL PROCEDURE AND CARE

28. Keep mice inside the ICU chamber 24 hours after the surgery and carefully control their welfare. **Important:** Access to food and water must be facilitated, so it is highly recommended to put some gel food in a petri dish on the floor of the cage. If dehydration signs are observed, inject SC 1 ml of saline solution 0.9% (w/v).
29. Follow the post-operative treatment protocol described in this scientific article.
30. Monitor the suture, animal welfare and behavior daily and carefully throughout the entire experiment but especially in the first month after surgery. If necessary, re-apply antibiotic ointment or re-suture the incision.
31. Record the animal body weight every Monday until the end of the experiment.
32. Change the cage for a completely clean one with some environmental enrichment object (such as cardboard roll, paper wipe…) at least once a week until the end of the experiment.

V. REFERENCES

DeVos, SL and Miller, TT. Direct Intraventricular Delivery of Drugs to the Rodent Central Nervous System. *Journal of Visualized Experiments*, 2013. DOI: https://dx.doi.org/10.3791/50326

Coto-Vilcapoma, MA; Castilla-Silgado, J; Fernández-García, B; Pinto-Hernández, P; Cipriani, R; Capetillo-Zarate, E; Menéndez-González, M; Álvarez-Vega, M; Tomás-Zapico, C. 2022. New, Fully Implantable Device for Selective Clearance of CSF-Target Molecules: Proof of Concept in a Murine Model of Alzheimer's Disease. Int J Mol Sci, 2022, 23:9256. DOI: https://doi.org/10.3390/ijms23169256

-Supplementary Information S2-

Table 1. Specifications of the drugs used for analgesic, anti-inflammatory and antibiotic treatment.

	CAS No.	Final dose (mg/kg)	Brand/Company	Stock (mg/ml)
Buprenorphine	52485-79-7	0.05	Bupaq 0.3 mg/ml (Richter Pharma, Austria)	0.3
Meloxicam	71125-38-7	2	Metacam 5 mg/ml (Boehringer Ingelheim, Germany)	5
Enrofloxacin	93106-60-6	10	Syvaquinol 25 mg/ml (Syva Laboratories, Spain)	25

Table 2. Volume (μl) of drug to be administered according to animal body weight (g)

Animal Body Weight (g)	Buprenorphine 0.05 mg/kg (μl)	Enrofloxacin 10 mg/kg (μl)	Meloxicam 2 mg/kg (μl)
20	66.6	80	80
27.5	91.6	110	110
30	100.0	120	120
32.5	108.3	130	130
35	116.6	140	140

Same day of surgery (volume to be administered are indicated in Table 2)

- Subcutaneous injection (S.C.):
  - Buprenorphine
  - Enrofloxacin
- Intraperitoneal injection (I.P.):
  - 1 mL sterile saline solution (0.9% NaCl)

Postoperative treatment (S.C.)

- Immediately after surgery:
  - Meloxicam
  - 1 mL sterile saline solution (0.9% NaCl) or glucose saline solution (0.9% NaCl + 5% Glucose)
- 12 hours after surgery:
  - Buprenorphine (10 μl less than the volume indicated in Table 2)
  - 1 mL sterile saline solution (0.9% NaCl) or glucose saline solution (0.9% NaCl + 5% Glucose)
- 24 hours after surgery:
  - Buprenorphine (1/2 of volume indicated in Table 2)
  - Meloxicam (10 μl less than the volume indicated in Table 2)
  - 600 μl sterile saline solution (0.9% NaCl) or glucose saline solution (0.9% NaCl + 5% Glucose)
- 36 hours after surgery:
  - Buprenorphine (1/4 of volume indicated in Table 2)
  - 500 μl sterile saline solution (0.9% NaCl) or glucose saline solution (0.9% NaCl + 5% Glucose)
- 48 hours after surgery:
  - Enrofloxacin (1/2 of volume indicated in Table 2)
  - Meloxicam (10 μl more than the volume indicated in Table 2)
  - 500 μl sterile saline solution (0.9% NaCl) or glucose saline solution (0.9% NaCl + 5% Glucose)
- 72 hours after surgery (Third post-surgery day):
  - Enrofloxacin (1/2 of volume indicated in Table 2)
  - Meloxicam (10 μl more than the volume indicated in Table 2)
  - 500 μl sterile saline solution (0.9% NaCl) or glucose saline solution (0.9% NaCl + 5% Glucose)
- 96 hours after surgery (Fourth post-surgery day):
  - Enrofloxacin (1/2 of volume indicated in Table 2)
  - 500 μl sterile saline solution (0.9% NaCl) or glucose saline solution (0.9% NaCl + 5% Glucose)
- Second postoperative week:
  - Enrofloxacin (1/4 of volume indicated in Table 2)
  - 500 μl sterile saline solution (0.9% NaCl) or glucose saline solution (0.9% NaCl + 5% Glucose)

-Supplementary Information S3-

PROCEDURE AND POST-OPERATIVE MONITORING FORM

PI:	Surgical Procedure:
Surgeon(s):	Date(s) of Surgery:
Contact Phone:	Contact Email:
Cage Card/Animal IDs:	Bioethics Commission Report ID:

**LIST ANESTHETIC/ANALGESIC REGIMEN** *(add as many rows as needed)*			
Animal ID	Weight (g)	Drugs Administered (*Agent/Dose/Volume/Route*)	Time Given

ANESTHETIC MONITORING AND PERI-OPERATIVE CARE

- ☐ Anesthetic depth assessed with toe pinch and observation of respirations prior surgery and during surgery.
- ☐ Heating device used to maintain body temperature (Mean Temperature during surgery:__________)
- ☐ Ophthalmic ointment applied.

SURGICAL COMMENTS AND TIME ANIMAL RECOVERED AND RETURNED TO COLONY:

**POST-OPERATIVE MONITORING**<br>Follow-up MUST be recorded each day for the first and/or second week after surgery and every 3-4 days until endpoint<br>*(add as many rows as needed)*				
Date	Animal ID/ Body weight (g)	Condition Score*	Observations/Comments	Initials

***Condition score based on welfare scoresheet**

**ENDPOINT**				
Animal ID	Weight (g)	Drugs Administered (*Agent/Dose/Volume/Route*)	Euthanasia/Sacrifice Method	Humane Endpoint (YES/NO)
